# Mechanical Properties and Corrosion Behavior of Thermally Treated Ti-6Al-7Nb Dental Alloy

**DOI:** 10.3390/ma15113813

**Published:** 2022-05-27

**Authors:** Iosif Hulka, Nestor R. Florido-Suarez, Julia C. Mirza-Rosca, Adriana Saceleanu

**Affiliations:** 1 Department of Mechanical Engineering, Las Palmas de Gran Canaria University, 35017 Las Palmas de Gran Canaria, Spain; hulka.iosif@ulpgc.es (I.H.); nestor.florido@ulpgc.es (N.R.F.-S.); 2 Medicine Faculty, Lucian Blaga University of Sibiu, 550024 Sibiu, Romania; adriana.saceleanu@ulbsibiu.ro

**Keywords:** heat treatment, dental alloy, Ti-6Al-7Nb, corrosion resistance

## Abstract

Ti and its alloys have the most satisfactory properties for biomedical applications due to their specific strength, high corrosion resistance, and high biocompatibility. Ti-6Al-7Nb has been approved for clinical use, proving to be a viable replacement for the Ti-6Al-4V alloy that has been used for many decades in medical applications. In our study, the Ti-6Al-7Nb alloy underwent heat treatment, was cooled in various cooling media such as mineral oil and water, and was then quenched in the oven. The microstructure was investigated, and the mechanical characterization was carried out by Vickers microhardness test. Young’s modulus measurements and tensile tests were performed in order to study the effect of cooling media on the material. To study the corrosion behavior, in vitro studies were performed on the Ti-6Al-7Nb samples in simulated body conditions by using artificial saliva. It was observed that the martensitic phase changed as a function of cooling media, and a less intensive cooling medium decreases strength properties’ indicators as well as hardness values. The results emphasize that the use of heat treatment improves the passive layer’s resistance in the presence of artificial saliva.

## 1. Introduction

Biomaterials are of tremendous importance to mankind since they are used to replace diseased biological structures [1]. The most important requirement for a biomaterial is to be accepted by the human body, followed by adequate mechanical strength, good wear, and excellent corrosion resistance in a highly corrosive environment generated by the human body [2]. The materials most commonly used for the manufacturing of biomaterials include pure Ti, Ti-alloys, Ti–Al–V, Co–Cr alloys, and 316 L stainless steel [3]. Among biocompatible metal alloys, Ti and its alloys have the most satisfactory properties for biomedical applications due to their specific strength, high corrosion resistance, and high biocompatibility [4]. The further development of Ti-based alloys with enhanced properties for medical applications is a challenge nowadays and has been specifically studied by researchers [5]. Among the Ti-based alloys, Ti-6Al-4V is the most widely used; however, recently it was reported that it has significant toxicity on the human body, leading to health problems. To overcome this issue, new titanium alloys with non-toxic elements such as Nb, Zr, Ta, Mo, Fe, etc. have been developed. Among these alloys, Ti-6Al-7Nb has been approved for clinical use, proving to be a viable replacement for the Ti-6Al-4V alloy that has been used for many decades for medical applications [6]. Niobium is a non-toxic element and does not cause adverse reactions in the human body and also, when alloyed, decreases the modulus of the elasticity of titanium by bringing it closer to that of the bone. Titanium in its pure state at room temperature has a hexagonal close-packed structure (α phase) that experiences an allotropic conversion at 882 °C, transitioning to a body-centered cubic crystal structure (β phase). This transformation offers the possibility to obtain alloys with crystalline structures such as α, α + β, or β, depending on the alloying elements that stabilize the respective phase. The elements that stabilize the α phase increase the temperature at which the phase α is stable. Aluminum is one of them. The elements that stabilize the β phase, such as Nb, are those that allow it to be stable at temperatures below the transformation temperature or to form eutectoid systems with Ti. The studied Ti-6A1-7Nb alloy has an α + β structure since aluminum stabilizes the α phase, while Nb allows the β phase to be stable at temperatures lower than the transformation temperature. However, when used as an implant, like any other biomaterial, it is prone to failure. The reasons why a biomaterial fails are numerous, and they are related to mechanical, chemical, wear, manufacturing, and surgical issues [2]. Among these issues, the failure of implants due to corrosion is one of the demanding clinical issues, and it was studied by many researchers [7]. Corrosion is one of the main processes that causes problems when metals are used as implants in the human body. Body fluids contain water, dissolved oxygen, proteins, and ions, such as chlorine and hydroxides. As a result, the human body represents a highly aggressive environment for the implant. Thus, corrosion is an important factor when a metallic alloy is designed for in vivo service. Cytotoxic, carcinogenic, or allergenic ions may be released into the body if the corrosion process starts [8]. The types of corrosion in metallic implants are stress corrosion cracking and fatigue corrosion, which occur when the material in a corrosive environment is subjected to external mechanical stresses such as static or cyclic [9]. Mechanical stress causes the appearance of anodic areas where there is a greater intensity of tension, concerning the unstressed areas, which would behave as cathodic zones. Another important type of corrosion in implants is produced by wear. When materials are subjected to friction and the creation of anodic and cathodic zones, the destruction of the protective oxide layers of the implant can form fine debris particles that end up in living tissues. Therefore, metallic alloys used as biomaterials must be subjected to corrosion testing. In order to increase the corrosion resistance, titanium alloys are artificially hardened through anodizing and heat treatments. This is possible due to the existence of an allotropic transformation that might occur in the alloy and allows it to carry out the thermal treatments. It is well known that Ti alloys are nearly inert because of the passive oxide film that forms on its surface, protecting the implant against corrosion attack. However, when subjected to body fluids and taking overloads of replaced biological structures, the thin oxide layer might fail, and thus unwanted metal ions would be released into the body. 

Thus, we aimed to study the effect of heat treatment followed by cooling in various media on titanium alloys in order to improve biocompatibility by enhancing the corrosion resistance of the protective layer of oxides developed on the surface of the samples; the hardening effect on the martensitic phase was studied as well. The mechanical properties were studied by microhardness and Young’s modulus measurements, as well as tensile test. In vitro studies on the Ti–Al–Nb biomaterials were performed in simulated body conditions by using artificial saliva. The effect of heat treatment followed by cooling in different media on the microstructural, mechanical characteristics, and corrosion behavior of Ti-6Al-7Nb can be of interest to the research community of biomaterials.

## 2. Materials and Methods

### 2.1. Sample Preparation

The Ti-6Al-7Nb alloy investigated in the present study was prepared in a double electronic flow melting oven, EMO 80 (ZIROM S.A., Giurgiu, Romania), using a vacuum value of 800–1100 Pa and a melting time of 55 min. The chemical composition of the alloy is presented in Table 1.

**Figure 1:**
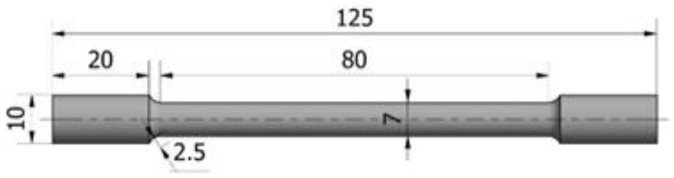
Dimension of tensile test specimens.

Cylindrical specimens were machined with the dimensions presented in Figure 1 prior to heat treatment. The specimens were not prepared according to standards; thus, the dimensions were selected only for laboratory testing purposes by the shape of ingots obtained after solidification in the furnace. 

### 2.2. Heat Treatment

The machined samples were heat-treated in argon atmosphere at 1150 °C (a rate of 5 °C/min was used) and then kept at this temperature for 100 min, followed by cooling in different media as: mineral oil, water, and the furnace. The treated samples were compared with a sample that had not undergone thermal treatment. The heat treatment followed by quenching influenced the physical–chemical properties of the samples and the development of the oxide layer on their surface.

### 2.3. Microscopic Observations

The Standard Guide for Preparation of Metallographic Specimens, ASTM E3-11 (2017) was used to perform the metallographic investigations. The samples were polished with emery paper from 280 to 1000 grit size and then with 0.1 µm alumina paste until a mirror-like surface was reached. Afterward, the samples were cleaned with acetone in an ultrasonic cleaner followed by etching with Kroll agent (10 mL HF, 5 mL HNO_3_, and 85 mL water). The prepared samples were investigated using an Olympus PME 3-ADL (Olympus, Tokyo, Japan) metallographic microscope, and the morphology of the samples was investigated by scanning electron microscopy (FESEM Zeiss Sigma 300 VP, Carl Zeiss, Jena, Germany). The SEM micrograph was acquired in low-vacuum mode to avoid sample charging, at a cathode voltage of 15 kV and a working distance of about 10 mm. A backscattered electron detector (BSD) was used in order to reveal the phases within the samples by using Z contrast. 

### 2.4. Microhardness Measurements

The microhardness measurements were performed using a Buehler Micromet VD 5124 (Buehler, Lake Bluff, IL, USA) hardness tester with a Vickers indenter. Loads of 49, 98, 196, and 980 mN were used with a 15 s holding time as mentioned in ISO 14577-1:2015 Metallic materials—Instrumented indentation test for hardness and materials parameters—Part 1: Test method. A Fisher scope H100 ultra micro hardness tester was used for measurements with very light loads of 50, 100, 200, and 1000 mN. The loads were applied gradually for 20 s, and afterward, the depth of indentation was measured; at the maximum value, a holding time of 15 s was maintained and gradually withdrawn for a corresponding time of 20 s. Based on the measurements, the elastic modulus was determined according to standard VDI/VDE 2616-1:2021, Hardness testing of metallic materials.

### 2.5. Tensile Strength Measurements

The tensile test was performed at room temperature using a Universal Testing Machine (Sistemas de Ensayos S.L., Madrid, Spain) using a constant speed of 10 mm/min until specimen failure. Three samples were prepared for each case, and after the tests, the fractured surfaces of the samples were measured manually and investigated using a scanning electron microscope (SEM), FE-SEM Hitachi S4800 (Hitachi, Tokyo, Japan), to study their topography.

### 2.6. Electrochemical Impedance Spectroscopy

Electrochemical measurements were performed in a conventional three-electrode cell in which the saturated calomel electrode (SCE) was the reference electrode, a platinum foil was the counter electrode, and the Ti-6Al-7Nb with different cooling conditions was the studied working electrode (WE). The working samples were mechanized in a cylindrical shape with an open area of 0.8 cm^2^. A low-temperature solder-insulated copper wire was attached to one surface of each specimen. The samples were embedded with epoxy resin so that only the other flat surface came into contact with the solution. The flat surface of each sample was ground on graded emery paper to 2500 grid, and the final aspect was achieved on a polishing wheel using 0.3 µm alumina colloidal suspension. The smoothed surface was then cleaned thoroughly in ethanol prior to use and dried at ambient temperature. The solution used for electrochemical measurements was altered Fusayama synthetic saliva with the composition as indicated: 400 mg/L NaCl; 400 mg/L KCl; 800 mg/L CaCl_2_·H_2_O; 695 mg/L Na_2_H_2_PO_4_·H_2_O; 300 mg/L KSCN; 5 mg/L Na_2_S·9H_2_O; 1000 mg/L urea (CH_4_N_2_O). The tests were carried out using a BioLogic SP-150 Potentiostat (by EC-LAB computer-controlled, Biologic, Seyssinet-Pariset, France) operated by Potentio Electrochemical Impedance Spectroscopy (PEIS). The open corrosion potential (versus the reference electrode SCE) was monitored after immersion of the working electrode in the synthetic saliva for 24 h when the potential stabilized within ±1 mV. First, the linear polarization resistance measurements were performed by polarizing the samples in a potential range of ±25 mV vs. E_corr_, allowing the estimation of polarization resistance R_p_ values. Next, the polarization curves were recorded in a potential range of ±175 mV vs. open circuit potential in order to estimate the corrosion current using the Tafel extrapolation method. Electrochemical impedance spectroscopy testing was then performed; EIS data were collected for the Ti-6Al-7Nb samples at corrosion potentials (E_corr_). The frequency was scanned from 0.1 Hz 100 kHz, with a ±20 mV peak-to-peak AC waveform superimposed on a DC bias potential to obtain Nyquist and Bode plots. The optimal equivalent circuit of the EIS experimental data was estimated by the fit-and-simulate procedure. For each experimental condition, three to five readings were taken to guarantee the accuracy and repeatability of the results. The standard used was ISO 16773-1-4:2016, Electrochemical impedance spectroscopy (EIS) on coated and uncoated metallic specimens.

## 3. Results and Discussion

### 3.1. Microstructure

**Figure 2:**
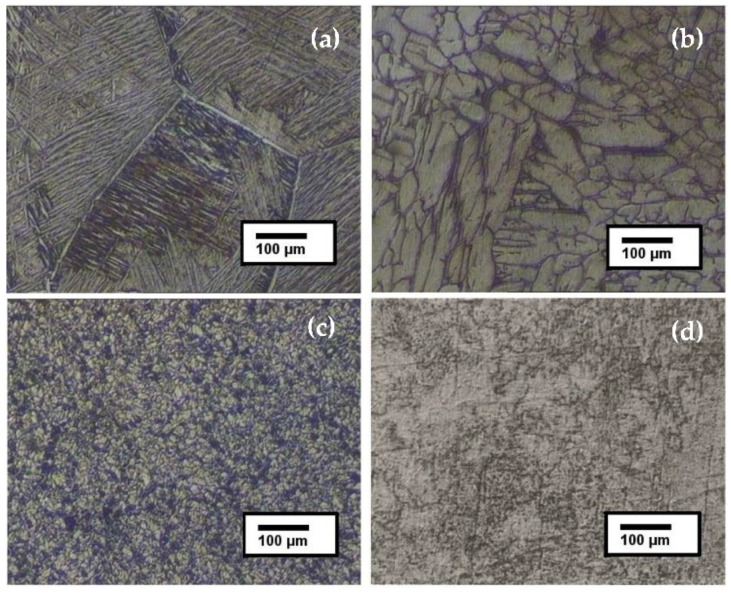
Microstructure of heat-treated Ti-6Al-7Nb alloy: (**a**) quenched in mineral oil, (**b**) quenched in the oven, (**c**) quenched in water, and (**d**) without treatment.

To obtain α–β phase microstructures, it is necessary to bring the material to temperatures higher than the beta transus temperature where the homogenization in the β phase takes place, followed by recrystallization in the α–β field, and finally, aging at lower temperatures to end the process [10]. The optical microstructures of Ti-6Al-7Nb heat-treated samples cooled in different media and compared with the non-treated sample are presented in Figure 2. One can see that the grain shape changed significantly when using different cooling media after the heat treatment. In the micrographs, the β phase is represented by dark areas, while the α phase is represented by gray areas. For the non-treated sample, a biphasic structure formed by α and β phases can be seen with β-grain boundaries barely visible similar to the observations reported by Paradkar et al. [11]. At 1150 °C, the material was in the β-phase region; thus, when performing the heat treatment and quenching, the sample presented a structure formed with a new α phase which began to form below the β transus temperature [12]. Sample 1, cooled in mineral oil (Figure 2a), presented a martensitic acicular phase formed from the previous β grains. From the micrograph, one can observe the remarkable increase in the grain size of the material as a consequence of the heating in the β region and cooling somewhat slower compared with water quenching. The growth of the martensitic phase was massive, characterized by the formation of parallel acicular phases when cooling from the homogenization step within the β phase. Using oil for quenching enabled the formation of α phase colonies with acicular structure and a continuous α-phase layer at the grain boundaries, according to the findings of G. Lutjering in Ti alloys [10]. Sample 2, annealed in the oven (Figure 2b), presented a structure made up of columnar alpha, equiaxial alpha, and intergranular beta phases. Similar morphology was obtained by Jovanović et al. [13] when furnace-cooling was used after heat treatment on a Ti–6Al–4V alloy. The difference in grain morphology compared with Sample 1 was attributed to the cooling time, which took longer in this case, and thus the crystallization took place more slowly. Sample 3, cooled in water (Figure 2c), presented fine equiaxed α and β grains, indicating a rapid cooling process. The structure obtained after quenching in water indicated a biphasic structure with an increased α phase, according to the microstructure presented in Figure 2a, cooled in mineral oil.

**Figure 3:**
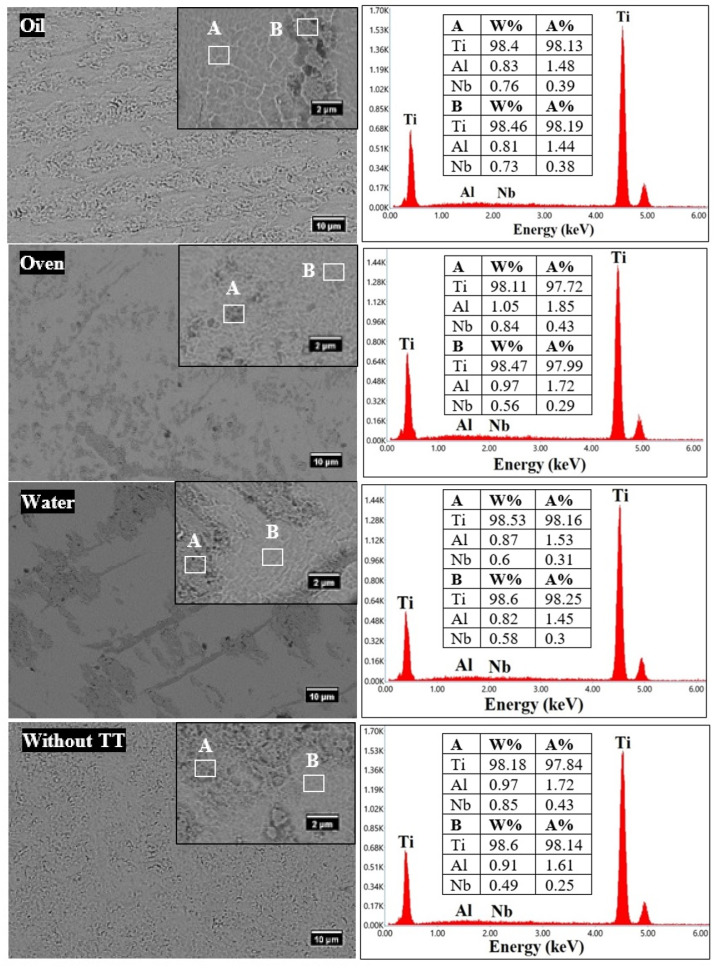
SEM micrographs at 1000× magnification and at 5000× magnification (upper right corner), EDX spectra, and quantification.

As can be seen in Figure 3, the alloys presented two phases with different distributions. Besides the alpha and beta phases, no other phases were identified within the alloys, and thus the results are in accordance with the micrographs collected on the metallographic microscope. The omega phase, shown experimentally to be formed by cooling certain metastable beta–Ti alloys [14,15,16], was not identified. The alpha phase is represented by gray areas (which are smoother), and the beta phase is represented by dark gray areas that look coarser. The difference between the two phases was attributed to the backscattered detector (BSD) used during the SEM investigations, as well as to the Kroll’s etchant used to reveal the phases. Since the EDX spectra were similar for both phases, only the one representative for the alpha phase is presented. Thus, the quantification results are presented in tables for the investigated areas of each sample.

### 3.2. Microhardness and Elastic Modulus

**Figure 4:**
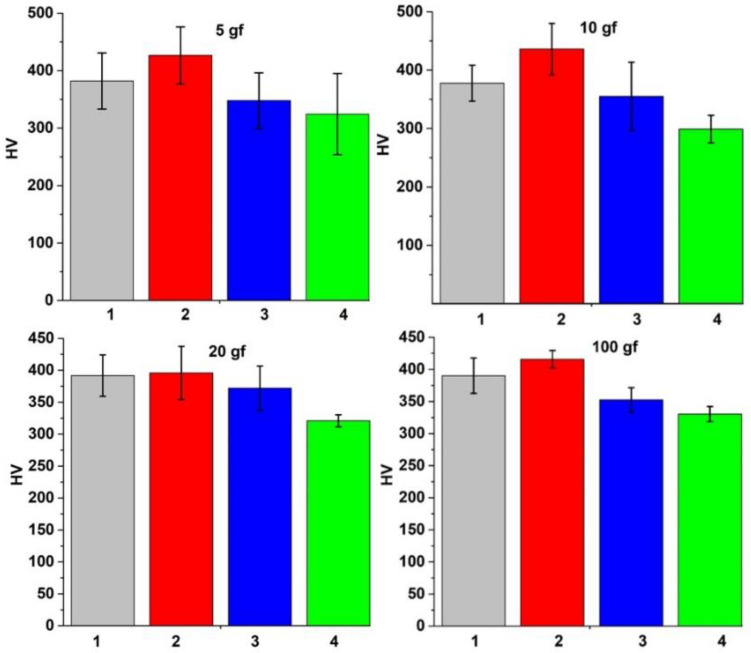
HV measurements of samples: (1) quenched in oil, (2) quenched in water, (3) without thermal treatment, and (4) quenched in oven.

A large dispersion of individual values was obtained for each sample with the applied loads, which was attributed to the different hardness values of the α and β phases as well as the crystal orientation within the material [17]. Ten measurements were made with each testing load to minimize these position-dependent factors, and their average values are presented and compared in Figure 4. The mechanical behavior was significantly affected by the cooling media, which influenced the formation of the new α phase after quenching. A minimum hardness value of 280 HV and an elastic modulus of 108 GPa were obtained for a mixture of α–β phase with a high content of β phase (sample quenched in the oven under 100 mN load), and a 505 HV hardness with 145 GPa modulus of elasticity was obtained for the sample with the higher content of α phase (sample quenched in water under 100 mN load).

**Figure 5:**
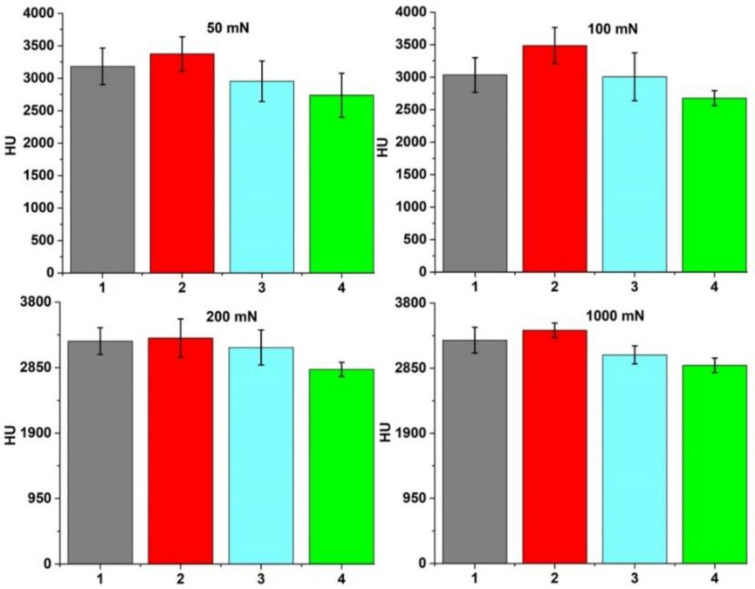
Ultra HV measurements of samples: (1) quenched in oil, (2) quenched in water, (3) without thermal treatment, and (4) quenched in the oven.

The Ultra HV measurements performed under very light loads indicated results in accordance with the hardness measurements presented and described previously, as can be observed in Figure 5. Thus, the results are directly influenced by the α and β phase variations within the investigated samples.

The microhardness of all heat-treated samples had increased values compared with the non-treated sample due to the increase of α martensite, while the β-phase fraction decreased. The α was harder than the β phase, leading to an increased hardness value for the treated samples [18].

**Figure 6:**
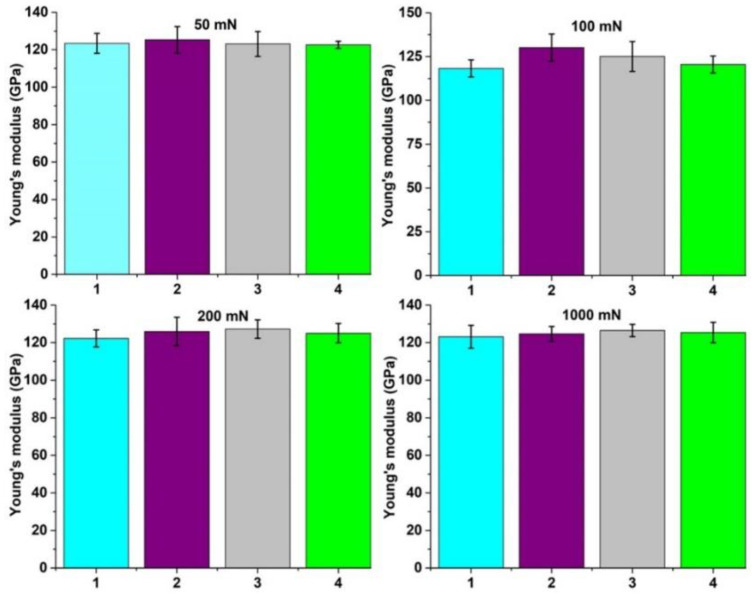
Elastic modulus of samples: (1) quenched in the oven, (2) quenched in water, (3) quenched in oil, and (4) without thermal treatment.

Elastic modulus values were calculated based on hardness measurements, and the results are plotted in Figure 6 as a function of the applied load. The results show that the values are similar to the non-treated sample, indicating that the heat treatment followed by cooling in various media did not significantly affect the elastic modulus. Overall, the values were lower than that of 316 L stainless steel used as a dental implant, which has a Young´s modulus of about 190 GPa; thus, the obtained values are closer to the human bone [2]. However, some differences might be noticed among the treated samples when applying loads of 100 mN and 200 mN. Among the samples, the one quenched in mineral oil presented lower values due to a lower hardness value attributed to the abundance of a softer phase within the structure. The microhardness and ultra microhardness results confirm the biphasic structure of the alloy: an α phase, hard with a high modulus of elasticity, and another β phase, which is softer and has a lower modulus of elasticity, confirmed by Borborema et al. [4].

### 3.3. Tensile Strength

The initial and calculated tensile test parameters are presented in Table 2. The initial length (L_o_), sample diameter (d_o_), and the surface area (S_o_), along with the maximum force at breaking (F_m_), were used to calculate the tensile strength σ_max_.

**Figure 7:**
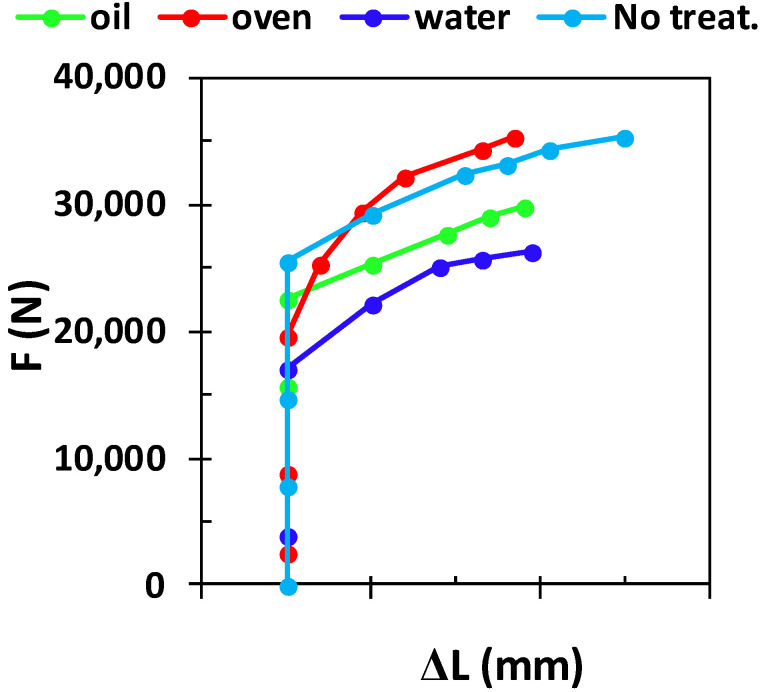
Force-elongation curves of tested samples.

Figure 7 presents the force-elongation curves obtained for the tested samples when applying a load until sample failure. One can see that the heat treatment followed by quenching affected the toughness of the samples, and thus all the samples became more fragile, compared with the toughness of the non-treated sample. Due to the presence of a higher amount of α phase, the sample quenched in water failed first at a force which was about 2700 N. When oil was used instead of water, the mechanical properties slightly decreased, in contrast to the non-treated sample. The non-treated sample presented a structure with a higher content of β phase, and thus it withstood a higher elongation before failure, in contrast to the sample quenched in the oven.

**Figure 8:**
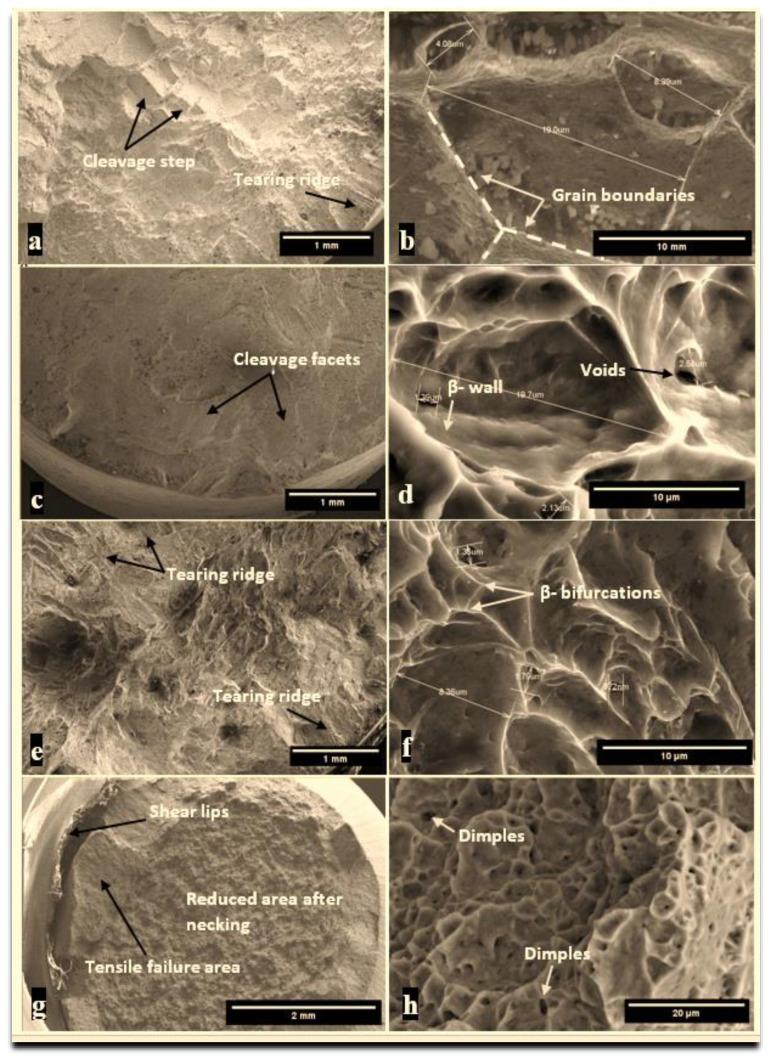
SEM microstructure of fractured surfaces of quenched samples in: oven (**a**,**b**), water (**c**,**d**), oil (**e**,**f**), and without thermal treatment (**g**,**h**).

The microstructure of fractured surfaces after tensile tests is presented in Figure 8. One can see that the heat-treated samples broke without elongation, which is the main characteristic of brittle fracture. By lowering the temperature below the transition temperature, the material changed from ductile to brittle. In our study, for all the heat-treated samples, the main fracture mechanism was a mixture of ductile and brittle fractures with dimples and cleavage-like morphology, while in the non-treated sample, the ductile fracture mechanism was predominantly distinguished by the formation of dimples with different shapes and sizes. Similar observations were reported by Zheng et al. when studying the effect of heat treatment on the mechanical properties of Ti alloys [19]. The fracture surfaces of samples quenched in the oven and oil were more tortuous compared with the surface of the sample quenched in water, which was flatter. The fractured surface of the sample quenched in water analyzed at a lower magnification indicated that the sample broke in a transgranular manner with surfaces perpendicular to the direction of the applied tension load. Generally, cleavage is the main cause of brittle fractures due to the breakdown of the atomic bonds between atoms found on two neighboring planes that are perpendicular to the direction of the applied force [20]. The microstructure of the non-treated sample was typical for ductile materials with a high degree of plastic deformation, indicating a rough surface topography. Compared with the heat-treated samples, one explanation for the differences in the elongation at break was the existence of a higher amount of β phase. An increased amount of β titanium improved the ductile behavior of the alloy [21]. This was also confirmed by the SEM micrograph, which showed the reduced area of the surface after necking. The dimple-fractured surface presented in the high magnification micrographs of the non-treated sample suggested a failure mechanism by void growth to coalescence, or microvoid coalescence. The dimples might have been traces of voids formed at inclusions or second-phase particles, as other authors reported [22]. Analyzing and comparing the fractured surfaces at higher magnification indicated that the heat-treated samples had a similar microstructure, regardless of the cooling media. Thus, there were differences in terms of grain size. Due to heat treatment, microstructural changes occurred, and thus the overall grain size increased, compared with the size of the non-treated sample. It can be seen that the elongation at which the samples were subjected during the tensile test changed the shape and size of the grains, compared with our findings after the optical microscopy investigations. According to the measurements performed on several SEM micrographs, it was observed that the larger grains were attributed to the sample cooled in the oven with an average length of about 20 μm, followed by the sample cooled in oil, with an average length of grains in the range of 4–19 μm. The finest structures were attributed to the sample cooled in water, with an average length of grains of about 8 μm.

### 3.4. EIS

**Figure 9:**
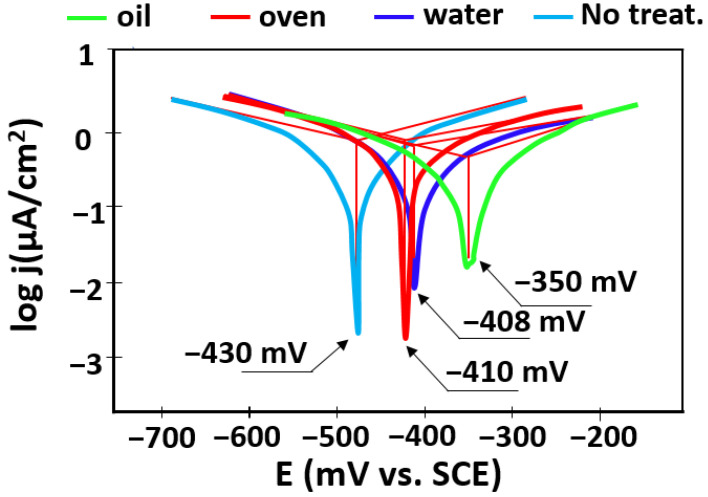
Polarization curves of the tested specimens.

The polarization curves of the tested specimens were recorded in the range of ± 175 mV vs. open circuit potential, plotted in semi-logarithmic coordinates (Evans diagrams), and were presented in Figure 9; as can be observed, the polarization curves had a similar behavior. Using the Tafel extrapolation method, instantaneous corrosion parameters in artificial saliva could be estimated and are presented in Table 3.

**Figure 10:**
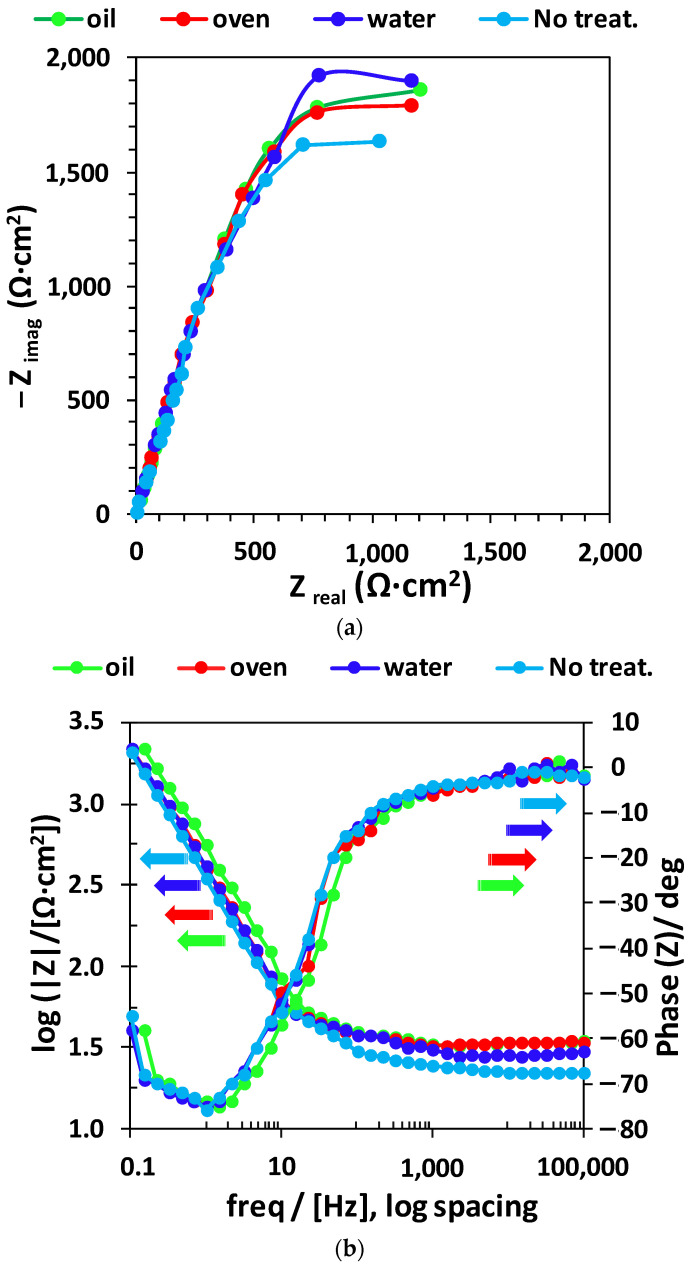
Nyquist (**a**) and Bode (**b**) diagrams of Ti-6Al-7Nb at 25 °C in artificial saliva and E_corr_.

The corrosion performance of titanium alloy samples in synthetic saliva was examined by EIS at ambient temperature, and the Bode and Nyquist graphs are plotted in Figure 10. From this figure, it can be observed that the shapes of the Nyquist plots (see Figure 10a) for the incoming and quenched samples were similar, showing a depressed semicircle. The Nyquist graphs did not generate perfect semicircles as would be predicted by EIS theory.

**Figure 11:**
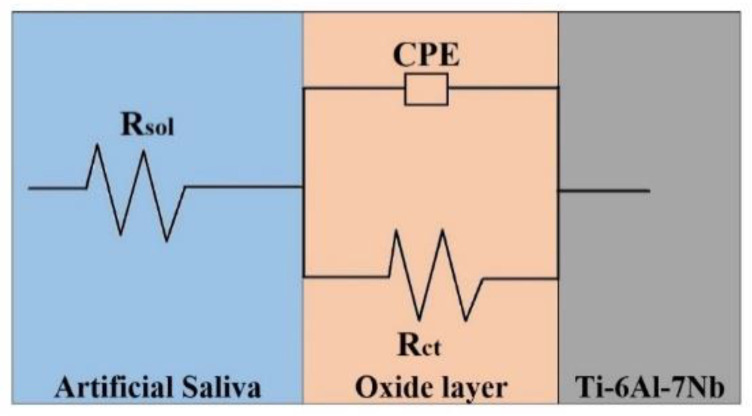
The electrical circuit used for the fit of experimental data.

The drift from the perfect semicircle was usually ascribed to frequency dispersion, as well as to the surface inhomogeneities and resistive mass transport [23]. From the diagrams, it is obvious that the impedance response of the untreated samples displayed a noticeable difference from that of the tempered specimens. The complete Nyquist impedance loop intersects the real axis at both higher and lower frequencies. At the high frequency point, the intersection matches the solution resistance (R_sol_), and at the low frequency point, it matches the sum of R_sol_ and the load transfer resistance (R_ct_). The difference of both values gives R_ct_. The R_ct_ value is a function of the electron transfer through the immersed area of the alloy and is inversely dependent with the corrosion rate [24]. The impedance response can be properly represented by using electrical circuits that can check and permit the calculation of numerical data corresponding to the physical and chemical characteristics of the electrochemical process under analysis [25]. The simple equivalent circuit presented in Figure 11 fits many electrochemical systems and consists of a parallel combination of a double-layer capacitance C_dl_ and R_ct_, corresponding with the corrosion process at the metal–electrolyte interface and R_sol_ between the studied alloy and reference electrode [26,27].

To minimize the effects caused by the metal surface’s unevenness, a constant phase element (CPE) was inserted into the circuit in place of a pure double-layer capacitance, which provided a more precise match [28]. The impedance of the CPE can be stated as Z_CPE_ = 1/ [Y^0^(jω)^n^], where Y^0^ is the magnitude of the CPE, n is the exponent (phase shift), ω is the angular frequency and j is the imaginary number. The CPE can be the resistor, capacitor, or inductor, depending on the magnitude of n [23,24,25]. Over the experiments, the obtained value of n oscillated between 0.82 and 0.99, which suggested a capacitive performance of the CPE. The values of EIS parameters, such as R_ct_, R_sol_ and CPE, are presented in Table 4, showing that the values of R_ct_ are increasing with the thermal treatment and the oil-quenched sample is showing the highest corrosion resistance. Chi-squared is the calculated sum of the weighted residuals and is a parameter indicating the goodness of fit. The chi-square values (χ^2^) for all data impedance were of magnitude 10^−4^, indicating an excellent agreement between the experimental data and the model using CPE in the fitting program.

The protective, passive film thickness increased in the following order: without treatment, water, oil, and oven. Such observations support the idea that the corrosion of titanium alloy was under the control of a charge transfer process [29]. The Bode spectra displayed in Figure 8 show the existence of a time constant, which corresponded with the depressed semicircle that was observed in the Nyquist diagrams.

## 4. Conclusions

In our study, the influence of the heat treatment followed by quenching in various media was aimed at optimizing the properties of Ti6A17Nb dental alloy in terms of structure, mechanical properties, and corrosion resistance in artificial saliva. The results were compared with a non-treated sample, and they led to the following conclusions:

- The microstructure of Ti-6Al-7Nb changed as a function of cooling media in terms of grain size and shape. Thus, the sample annealed in the oven presented a structure made up of columnar alpha, equiaxial alpha, and intergranular beta phases; the one cooled in water presented fine equiaxed α and β grains, indicating a rapid cooling process. For the sample annealed in oil, a remarkable increase was observed in the grain size of the material as a consequence of the heating in the β region and the cooling taking somewhat longer when compared with the water quenching. The mechanical tests showed that the values of elastic modulus were similar for all the studied samples, indicating that the heat treatment followed by cooling in various media did not significantly affect this parameter.
- For all the heat-treated samples, the main fracture mechanism was a mixture of ductile and brittle fractures with dimples and cleavage-like morphology, while in the non-treated sample, the ductile fracture mechanism was predominantly distinguished by the formation of dimples with different shapes and sizes.
- The polarization resistances showed very high polarization resistance in artificial saliva, and the obtained EIS spectra showed a compact passive film on the surface of the heat-treated samples. The oxide layer formed on the Ti6A17Nb specimens had the highest corrosion resistance in oil quenching (about 30% more compared with the non-treated sample).

## Figures and Tables

**Table 1:** Chemical composition of the Ti-6Al-7Nb alloy.

Composition (wt%)							
Al	Nb	Fe	C	O	N	Si	Ti
6.21	7.06	0.13	0.082	0.10	0.031	0.088	Balance

**Table 2:** Tensile test parameters.

Sample	L_o_ (mm)	d_o_ (mm)	S_o_ (mm2)	F_m_ (KN)	σ_max_ (MPa)
Oil	80	7	38.48	29.91	784.4
Water				26.23	681.8
Oven				35.30	927.1
No TT				35.30	927.1

**Table 3:** Electrochemical parameters obtained by the Tafel extrapolation method from polarization curves.

Sample	E_corr_ (V)	I_corr_ (μA/cm^2^)	β_c_ (V/dec)	β_a_ (V/dec)	R_p_ (kΩ)
Oil	−350	0.057	0.051	0.053	191.6
Oven	−410	0.059	0.036	0.041	182.5
Water	−408	0.063	0.049	0.037	163.7
No TT	−430	0.075	0.044	0.041	147.5

**Table 4:** Electrochemical parameters of equivalent circuits.

Sample	R_sol_ [Ω·cm^2^]	R_ct_ [Ω·cm^2^]	Y^0^·10^−6^ [Ω^−1^·cm^−2^·s^−n^]	n	χ^2^
Oil	33.88 ± 1.22	2754.22 ± 12.65	0.264 ± 0.032	0.84 ± 0.11	1.2 × 10^−4^
Water	30.19 ± 0.56	2187.76 ± 18.34	0.302 ± 0.065	0.84 ± 0.08	1.1 × 10^−4^
Oven	33.88 ± 1.03	2187.76 ± 11.12	0.133 ± 0.031	0.95 ± 0.05	2.3 × 10^−4^
No TT	21.87 ± 0.38	2089.29 ± 17.62	0.424 ± 0.062	0.96 ± 0.02	1.5 × 10^−4^
